# Supplementary material for: In Vivo Evaluation of Bone Regenerative Capacity of the Novel Nanobiomaterial: β-Tricalcium Phosphate Polylactic Acid-co-Glycolide (β-TCP/PLLA/PGA) for Use in Maxillofacial Bone Defects
Source: Nanomaterials (Basel). 2023 Dec 28;14(1):91. doi: 10.3390/nano14010091 (PMC10780666; doi:10.3390/nano14010091)
Supplement: Supplementary file 1 [file nanomaterials-14-00091-s001.zip › nanomaterials-2772169-supplementary.pdf]

Supplemental figures

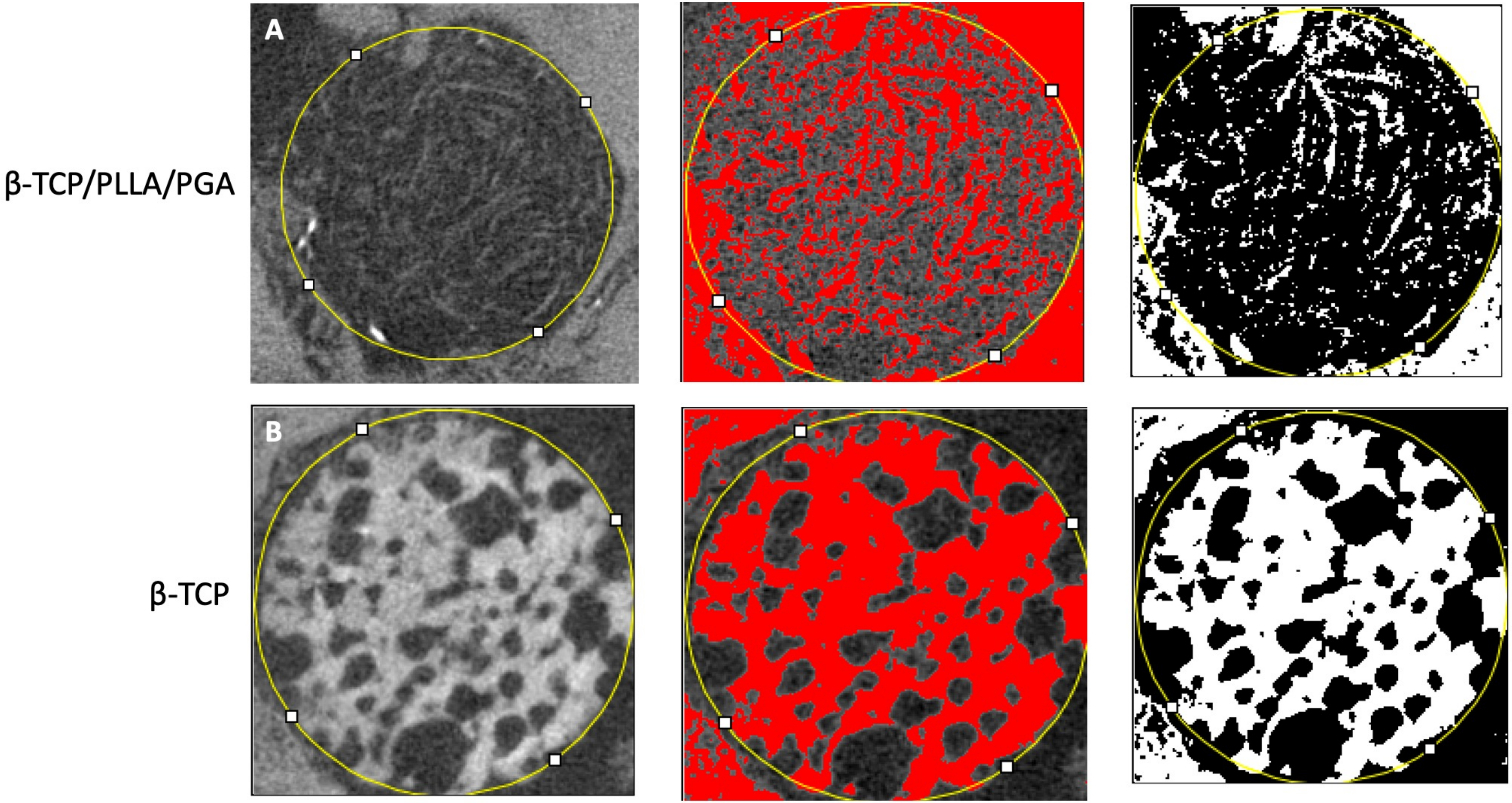

S1 - Redefining 4-mm critical size defect and Binary images of A: β-TCP/PLLA/PGA and B: β-TCP used for BV/TV calculation using ImageJ.

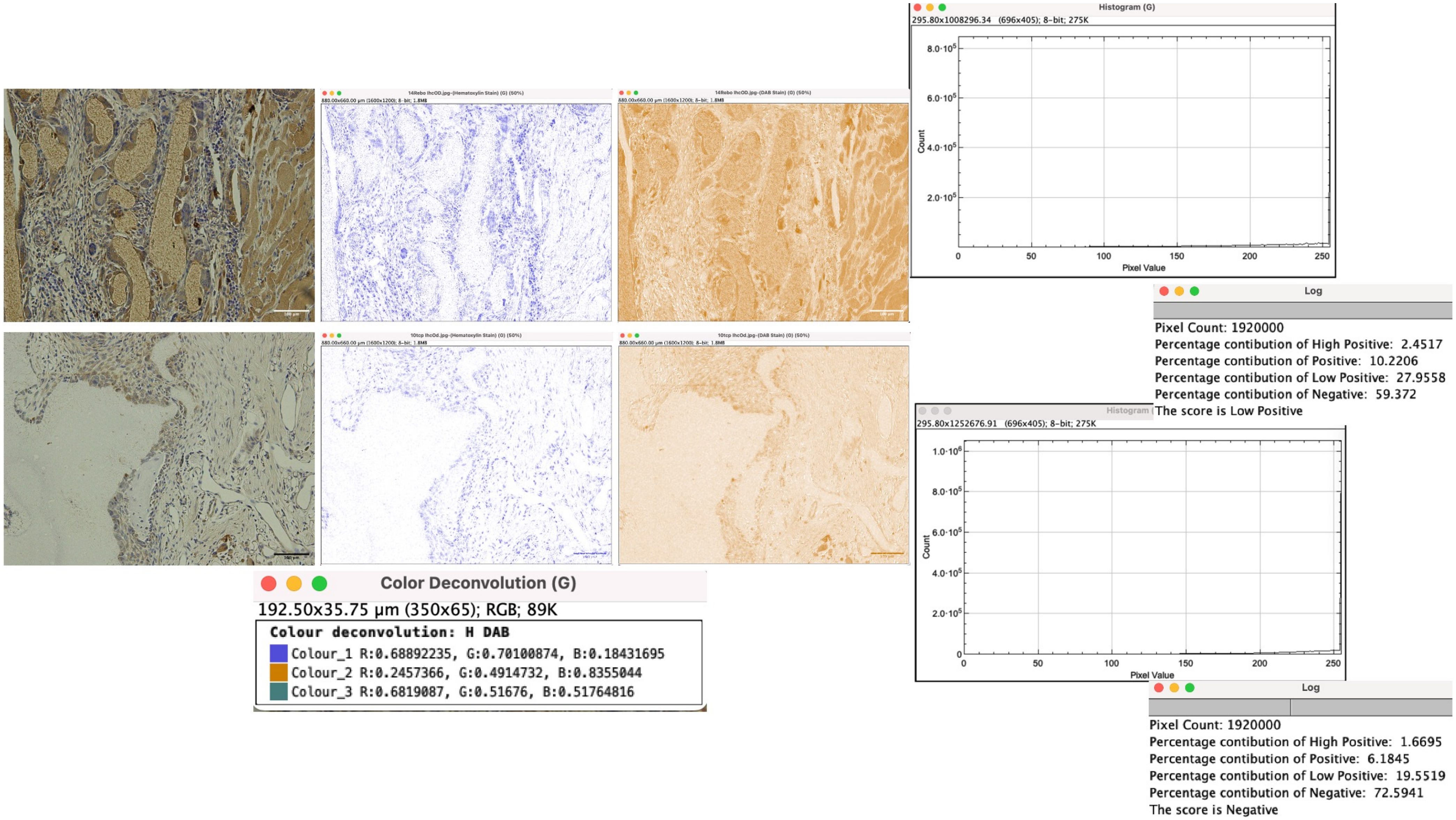

S2 - Method employed for IHC OD evaluation using ImageJ. β-TCP/PLLA/PGA (upper left to right) and β-TCP (lower left to right). Log displaying the score of the example image.
